# The Compensation of Veterinarians for Directing Public Work

**Published:** 1903-01

**Authors:** 


### THE COMPENSATION OF VETERINARIANS FOR DIRECTING PUBLIC WORK.

The salary of the President of the Pennsylvania Railroad is $50,000 a year. Several Vice-Presidents and the General Manager receive $25,000 per year, while salaries of from $6000 to $15,000 are numerous. The capitalization of the Pennsylvania Railroad, at present, is about $400,000,000, and it is to guard and advance a business represented by these figures that the salaries just enumerated are paid. Many bank presidents and officers of corporations doing a business that is very small, as compared to that of a railroad system, receive salaries quite as large as those cited.

The live-stock business is the greatest in the United States, and supports more families than any other. At the Chicago stockyards the volume of business last year amounted to more than $300,000,000. The total investment in live-stock and in equipments contributory to the live-stock industry amounts to much more than $6,000,000,000. To guard and advance the interests representing this astounding aggregation of capital, the United States Government has established the Bureau of Animal Industry. The prosperity of animal husbandry depends very largely upon the efficiency of this bureau. The chief of the Bureau of Animal Industry is charged with the responsibility of keeping the land free from animal plagues, of making foreign markets possible for our animals and animal products, of preventing the spread of indigenous plagues, and of protecting the health of the consumers of animal products. He is also charged with work calculated to encourage the improvement of domestic animals, and to render their production less costly. As compared to these duties, those of a president of a bank or a railroad are insignificant. There are plenty employees of corporations capable of filling the highest offices; there is only one man capable of filling the position of Chief of the Bureau of Animal Industry as well as it is now filled. Judged by the standards of commercial life, the Chief of the Bureau of Animal Industry should receive a salary of at least $50,000 a year. This is the salary of the President and to suggest it for the head of a subordinate department is absurd, however important the work of that department may be. But, judged by non-commercial standards, judged by salaries the government is paying, Dr. Salmon should receive not less than $6000 per year. England, with interests much less important, pays her chief veterinarian £2000 per year. At present, relatively speaking, the Chief of the Bureau of Animal Industry is the poorest paid member of his department.

Salaries for veterinarians are too low all along the line. It may be, however, that this is largely the fault of veterinarians. They have not made themselves felt sufficiently and have not impressed the public with the necessity and importance of their work and the fact that their pay should be commensurate with existing standards in other professions.

Koch charged the British Government $30,000 a year for his services in connection with the study of rinderpest in South Africa. We are indebted to Prof. Koch for this wholesome precedent. He is not a veterinarian, but his charge was for veterinary work.

Where a given amount of capital is in jeopardy and a given number of persons are threatened, and where the danger can be removed or averted only by men who have the best mental balance and a professional equipment that can be acquired only by the expenditure of long years of study and much exertion and money, a certain harmony should exist in relation to the compensation of the experts employed—no matter whether the fundamental question is one of engineering, law, statecraft, finance, medicine, or comparative medicine.

Again we say, judged by existing government standards, the Chief of the Bureau of Animal Industry should be paid not less than $6000 per year.

